# Antioxidant Activity of Honey Bee Products

**DOI:** 10.3390/antiox14010064

**Published:** 2025-01-08

**Authors:** Ivana Tlak Gajger, Josipa Vlainić

**Affiliations:** 1 NRL for Honeybee Diseases, Faculty of Veterinary Medicine, University of Zagreb, Heinzelova 55, 10000 Zagreb, Croatia; 2 Institute Ruđer Bošković, Bijenička Cesta 54, 10000 Zagreb, Croatia

Antioxidants have gained significant importance in modern nutrition. They can be sourced from various natural products, including those from bees [1]. Honey bee products are royal jelly, venom, beeswax, honey, propolis, pollen, and fermented pollen (bee bread). These substances are known for their antimicrobial, anti-inflammatory, antitumor, antioxidant properties, and different biological activities [2]. Throughout history, they have been used for various apitherapy purposes and are highly valued for their medicinal, cosmetic, and nutritional benefits [3,4]. These products are rich in antioxidants, which play a crucial role in combating oxidative stress linked to chronic diseases like cancer and cardiovascular conditions [5]. This Special Issue, entitled “Antioxidant Activity of Honey Bee Products”, focuses on the antioxidant properties of these products and their associated health benefits.

Honey is especially noteworthy for its high levels of phenolic compounds and flavonoids [6,7,8], all of which exhibit significant antioxidant activity. Propolis is similarly rich in flavonoids and phenolic acids, which contribute to its powerful antioxidant and anti-inflammatory properties [9,10]. Royal jelly, recognized for its protein-rich composition, contains several phenolic compounds that also demonstrate antioxidant effects [11]. While beeswax is not as commonly researched, it does show some mild antioxidant activity as well [12]. Also, pollen and bee venom are promising natural sources of antioxidants that may help prevent diseases related to oxidative stress, although more research is needed to explore their mechanisms and potential therapeutic applications [13,14,15]. The beneficial effects of honey bee products have been widely recognized in traditional medicine, and they show potential for contemporary therapeutic uses. Additionally, these products are often linked to health benefits and are considered valuable natural foods.

The primary focus of this Special Issue was to summarize previous findings, present new research, and align this information with current global health needs as remedies and agents that promote health, reducing the risk of various diseases. In particular, oxidative stress is a key factor in many modern diseases, and honey bee products may provide supportive benefits due to their antioxidant capacity. We aimed to discuss the bioaccessibility and bioavailability of these complex natural products, which can vary based on their composition and geographical origin. To enable accurate comparisons, it is essential to standardize their quality parameters and ensure that the same methods and measurement units are used across different matrices of bee products [16,17]. This Special Issue brings together three reviews and twelve original articles that explore recent research focused on enhancing the potential functionalities of various bioactive compounds, such as phytochemicals, found in bee products. These studies provide insights into the effectiveness of these compounds as natural alternatives for managing health conditions related to oxidative stress.

Bee pollen is increasingly recognized as a functional food because of its high-quality composition and therapeutic potential in medical and culinary applications. A review by El Ghouizi et al. (Contribution 1) highlights important details about the composition of bee pollen, particularly its phenolic compounds, as well as its biological properties and molecular pathways. Although its diverse composition provides various pharmacological benefits, the significant variability in its content poses a challenge to its use in phytomedicine. On this basis, more attention should be paid to standardization, including phenolic composition and nutritional value of different plant-origin pollen; to controls in the frame of production beekeeping practices; to more pharmacological and biochemical examinations; to enhance the bioavailability of bee pollen bioactive compounds; to the bio- and techno-functional value of bee pollen as food, such as novel bee pollen-enriched food products or dietary supplements; and to more clinical trials to investigate the beneficial effect of pollen as functional food on human and animal health.

Martiniakova et al. (Contribution 2) summarized the current animal and human studies regarding using honey as a potential therapeutic agent for osteoporosis and breast cancer which are posing significant socioeconomic challenges. Preclinical in vitro studies indicate that honey has a beneficial impact on bone health and breast tissue health where honey has a significant impact on the microstructure and strength of bones, as well as on oxidative stress. It also influences breast cancer by affecting cell proliferation, apoptosis, tumor growth rate, and volume. Clinical studies focused on breast cancer have shown that honey can effectively increase blood cell counts, interleukin-3 levels, and overall quality of patient life. These findings suggest that honey may serve as a promising therapeutic supplement for promoting the health of bone and breast tissue.

Honeybee products offer known pharmacological and health benefits, particularly concerning periodontal disorders, which are caused by dental biofilm and an inflammatory response to bacterial overgrowth resulting from dysbiosis in the oral microbiome. Choudhary et al. (Contribution 3) reviewed the potential role of these bee products in preventing oral diseases, highlighting their diverse biologically active compounds, including flavonoids, phenolic acids, and terpenoids. These findings suggest that bee products could serve as a therapeutic option for individuals suffering from various oral disorders.

Bae et al. (Contribution 4) propose that properly purified Korean-origin propolis could be used as a cosmetic ingredient that helps mitigate human skin toxicity caused by air pollutants. This effect is attributed to propolis hydrophilic and lipophilic fractions and several key bioactive components that influence the viability and oxidative stress of HaCaT epidermal keratinocytes exposed to particulates with a diameter smaller than 10 μm.

Honeybee products were assessed for their potential to protect against nephrotoxicity induced by doxorubicin. The combined treatment of honey, royal jelly, and propolis significantly improved biochemical, histological, and immunohistochemical parameters in rat renal tissue, leading to a notable enhancement in the expression of the PARP-1 and Bcl-2 genes (Contribution 5).

Moskwa et al. (Contribution 6) compared the chemical composition, total phenolic content, and concentration of toxic elements in Polish propolis extracts and New Zealand Manuka propolis extracts and evaluated their anticancer potential against diffuse astrocytoma derived from patient cells and glioblastoma cell lines (T98G, LN-18). Both propolis extracts showed antioxidant capacity and exhibited similar activities, demonstrating promising anti-glioma potential for in vitro experimental conditions. To support the authenticity of Polish honeybee varieties, phenolic acids were analyzed as indicators. Specifically, syringic acid, vanillic acid, and caffeic acid are characteristic of linden honey, while p-coumaric acid and 4-hydroxybenzoic acid are associated with buckwheat honey and vanillic acid is notable in honeydew honey. Of these, buckwheat honey has the highest median total phenolic content, indicating a rich concentration of phenolic compounds; thus, it is recommended for enriching human diets with its antioxidant ingredients (Contribution 7).

Kelulut honey has excellent antioxidative and anti-inflammatory properties and unique physicochemical characteristics. It is being studied for its isolated and combined effects with metformin or clomiphene in addressing oxidative stress and reproductive and metabolic abnormalities associated with polycystic ovary syndrome. The results indicate that this combination can improve oxidative stress, hormonal profiles, and the estrous cycle in rats, suggesting a potential complementary treatment option for women with this condition (Contribution 8).

Montaser et al. (Contribution 9) conducted chromatography on the HC2 fraction and found that the secondary metabolites in citrus honey and marjoram honey, specifically hesperetin, linalool, and caffeic acid, are responsible for increasing antioxidant activities in comparison with clover honey.

The color indexes of 39 propolis samples from various locations in Turkiye were determined for the first time using the Lovibond Tintometer. This study also examined the relationship between the color index, total phenolic content, and the cytotoxic and antioxidant activities of the propolis samples, including two commercial ones. The research highlighted how these samples can be characterized by their color indices, chemical contents, and potential activities, such as antioxidant, antiviral, and cytotoxic properties. These findings suggest that propolis could be useful in various fields, ranging from medicine to cosmetics (Contribution 10).

Zakaria et al. (Contribution 11) investigated the therapeutic effects of stingless bee (*Heterotrigona itama*) bee bread (fermented pollen) from Malaysia on obesity-related disorders in hepatic lipid metabolism. They focused on its Keap1/Nrf2 pathway regulation and proposed that it could serve as a natural supplement for treating obesity-related fatty liver disease.

Dimitriu et al. (Contribution 12) reported that honey enriched with polyphenols from raspberry extracts maintains the characteristic properties of honey while enhancing the synergistic antioxidative activity between honey and the polyphenols. Although a honey-biomimetic natural deep eutectic solvent, which has similar properties to honey, demonstrated comparable antioxidant activity when mixed with polyphenols, honey appears to possess additional qualities that further enhance synergism and reduce antagonism.

The total phenolic content, antioxidant activity, and phenolic compounds of honey from Western Australia were measured, and the results were ranked as follows: *Calothamnus* spp. (Red Bell) had the highest levels, followed by *Eucalyptus marginata* (Jarrah), *Agonis flexuosa* (Coastal Peppermint), and *Corymbia calophylla* (Marri). Similar trends were observed in their respective Ferric Reducing Antioxidant Power (FRAP) and 2,2-diphenyl-1-picrylhydrazyl (DPPH) antioxidant activities (Contribution 13).

Cerumen is a bee product made exclusively by stingless bees, composited as a mixture of beeswax and plant resins. Ferreira et al. (Contribution 14) investigated the chemical composition and antioxidant activity of cerumen produced by the Geotrigona sp. and Tetragonisca fiebrigi stingless bees. They conducted both in vitro and in vivo analyses using HPLC, GC, and ICP OES techniques. The results showed promising effects against oxidative stress and related diseases.

Various interactions within complex chemical systems influence the antioxidant and prebiotic properties of mixtures that combine honey with polyphenol-rich extracts. Typically, the modulation of antioxidant activities varies between the two extracts, which can be attributed to differences in the composition of polyphenols found in the tested plant extracts. Notably, the honeysuckle flower extract demonstrated higher prebiotic activity than the raspberry extract, and the effect on lactic acid production resulted in a hormetic response (Contribution 15).

The published scientific papers highlight the significance of utilizing the antioxidative properties found in various compounds of natural bee products. By exploring a diverse range of bioactive substances present in propolis, honey, pollen, cerumen, and other products, the authors are paving the way for sustainable beekeeping and apitherapeutic practices. Additionally, incorporating natural antioxidants into functional foods presents an innovative and promising opportunity to enhance humans’ and animals’ health and well-being. 
